# IFN-β signaling dampens microglia reactivity but does not prevent from light-induced retinal degeneration

**DOI:** 10.1016/j.bbrep.2020.100866

**Published:** 2020-11-26

**Authors:** Verena Behnke, Thomas Langmann

**Affiliations:** a Laboratory for Experimental Immunology of the Eye, Department of Ophthalmology, Faculty of Medicine and University Hospital Cologne, University of Cologne, 50931, Cologne, Germany; b Center for Molecular Medicine Cologne (CMMC), 50931, Cologne, Germany

**Keywords:** Microglia, Interferon-β, Light damage, Complement factors, Age-related macular degeneration

## Abstract

Chronic activation of microglia is associated with retinal degeneration, which makes them a potential therapeutic target for retinal degenerative diseases including age-related macular degeneration (AMD). Interferon-beta (IFN-β) is a potent immune regulator, commonly used for the treatment of multiple sclerosis patients. We have previously shown that IFN-β prevents microgliosis and choroidal neovascularization in a laser model of wet AMD. Here, we hypothesized that microglia modulation via IFN-β may also dampen mononuclear phagocyte reactivity and thereby protect from retinal degeneration in a light-damage paradigm mimicking some features of dry AMD. BALB/cJ mice received intraperitoneal injections of 10,000 U IFN-β or vehicle every other day; starting at the day of exposure to 15,000 lux white light for 1 h. Systemic treatment with IFN-β partially enhanced IFN-α/β receptor (IFNAR) signaling in the retina and reduced the number of reactivated microglia in the subretinal space. However, four days after light damage neither decreased expression of complement factors nor rescue of retinal thickness was found. We conclude that IFNAR signaling modulate retinal microglia but cannot prevent strong retinal degeneration as elicited by acute white light damage.

## Highlights

• Intraperitoneal application of IFN-β increases IFNAR signaling in the retina.
• IFN-β administration reduces microglia migration to the subretinal space.
• Light-induced retinal degeneration is not restored by IFN-β therapy.

## 1 Introduction

Age-related macular degeneration (AMD) is one of the leading causes of irreversible vision loss in the elderly of the western world. In terminal stages of its two forms, dry and wet, AMD can lead to complete vision loss that has a severe negative impact on the patient's quality of life. AMD is a multifactorial disease, where its main risk factors are advanced age, smoking, and variations in genes of the immune system, especially those regulating the complement system [1,2]. The number of affected people is rising rapidly due to the demographic change and additional therapy options are urgently needed.

Microglia are the resident immune cells of the central nervous system [3]. They build a regularly spaced network of ramified cells and are essential for homeostatic functions of the retina [4,5]. Microglia also play a key role in the initiation and perpetuation of chronic inflammation in the aging retina [6,7]. Furthermore, mononuclear phagocyte reactivity is associated with human AMD and related mouse models [[8], [9], [10]]. Therefore, signaling pathways and molecular targets that modulate microglia and macrophage activity represent attractive therapeutic treatment options [11]. Despite the fact that various animal studies highlighted the beneficial effects of immunomodulation [12], there are currently no clinically approved therapies targeting mononuclear phagocyte reactivity.

Interferons (IFNs) are endogenous cytokines that exhibit potent immunomodulatory, anti-viral and anti-proliferative properties, which makes them applicable for different clinical treatments [[13], [14], [15], [16]]. While IFN-α and -γ are promoting pro-inflammatory responses, IFN-β is mainly immuno-regulatory. All type I interferons connect to the ubiquitously expressed IFN-α dimer receptor composed of Interferon-α/β receptor 1 (IFNAR1) and IFNAR2. Receptor activation can induce the canonical JAK-STAT or alternative signaling pathways and their cooperative functions then result in a pleiotropic cellular, and thus, divergent biological response [17,18]. There is strong evidence that IFN-β has an immunomodulatory effect on microglia, and thereby counteracts autoimmunity in the central nervous system [19,20]. We have shown previously that IFN-β administration exerts a positive therapeutic effect in a laser model of wet AMD [21]. IFN-β treatment effectively attenuated microgliosis and macrophage response and reduced the area of choroidal neovascularization (CNV).

Here, we studied whether modulation of microglia via IFN-β may also dampen mononuclear phagocyte reactivity by altering the production of complement components in the retina, and thus, protect from retinal degeneration in a light-damage setting that mimics some features of dry AMD.

## 2 Material and methods

### 2.1 Cell culture

Murine BV-2 cells from passages 8–20 were used and cultured at 37 °C and 5% CO_2_ humidity in T75 flasks. Culture media RPMI 1640 (Gibco; Waltham, USA) contained 5% fetal bovine serum (FBS; Gibco; Waltham, USA), 1% Penicillin/Streptomycin (Gibco; Waltham, USA), 3 mM L-Glutamin (Gibco; Waltham, USA), and 50 μM β-Mercaptoethanol (Sigma-Aldrich; Darmstadt, Germany). Media was changed every 3 days and cells were split at a confluency of 95%. Human interferon-beta 1a was purchased from PeproTech GmbH (#300–02BC; Germany) and diluted in phosphate-buffered saline (PBS) with 0.1% BSA. BV-2 cells were cultured in 6 well plates, with 3*10^5^ cells per well and treated with 50 ng/mL Lipopolysaccharide (LPS-EB; InvivoGen; San Diego, USA) together with 1000 U/mL IFN-β or IFN-β alone for 6, 24, and 72 h. After treatments, media was removed and RNA isolation was performed.

### 2.2 Animals

8-10-week-old male and female BALB/cJ mice were used in the experiments [[21], [22], [23]]. The animals were kept under SPF-conditions in an air-conditioned environment with a 12 h light/dark cycle and water and food *ad libitum*. All experimental protocols complied with the ARRIVE guidelines and were carried out in accordance to the German animal welfare act, which is in line with the European Directive 2010/63/EU, and the ARVO Statement for the Use of Animals in Ophthalmic and Vision Research. The animal experiments used in this study were reviewed and approved by the governmental body responsible for animal welfare in the state of North Rhine-Westphalia, Germany (Landesamt für Natur, Umwelt und Verbraucherschutz; application no. 81–02.04.2017.A466).

### 2.3 Light exposure and IFN-β administration

Littermates were dark-adapted for 16 h prior to light exposure. Pupils were dilated with 2.5% phenylephrine and 1% tropicamide under dim red light and mice were placed separately in reflective, aluminum-foil-coated cages to prevent covering. Bright white light with an intensity of 15,000 lux for 1 h was applied. After light exposure, the animals were held in dark-reared conditions overnight, then transferred to normal light cycle until further analysis. Human interferon-beta 1a was purchased from PeproTech GmbH (#300–02BC; Germany) and diluted to 10,000 U/100 mL in PBS and 0.1% BSA. Intraperitoneal application of 10,000 U per mouse started 1 h prior to light damage and continued every other day until the end of the experiments.

### 2.4 *In vivo* imaging using spectral-domain optical coherence tomography (SD-OCT)

Retinal thickness was analyzed 4 days after light-damage using SD-OCT with the HRA/OCT device (Spectralis™, Heidelberg, Germany). Mice were anesthetized with Ketamin (Ketavet 100 mg/kg)/Xylazin (Rompun 10 mg/kg) and pupils were dilated with 2.5% phenylephrine and 1% tropicamide.

### 2.5 RNA isolation, reverse transcription and quantitative real-time polymerase chain reaction (q-RT-PCR)

RNA from cultured microglial cells was isolated with the RNA Isolation Kit following the manufacturer's instructions (Machery & Nagel; Düren, Germany). RNA from retinas was isolated with the Qiagen Micro isolation kit according to the manufacturer's protocol. Purity and integrity of the RNA was assessed with a NANODrop 2000 machine (Thermo Fisher Scientific; Waltham; USA). cDNA was synthesized with the Thermo Fischer Reverse Transcriptase Kit according to the company's protocol (Thermo Fisher Scientific; Waltham; USA). Subsequent qRT-PCR analysis was performed in duplicates with the Takyon Probe Assay protocol (Eurogentec Deutschland GmbH; Cologne, Germany) using the LightCycler®480 II (Roche; Basel, Switzerland). Primer sequences and Roche Universal Probe Library probe numbers were as follows *ATP synthase, H+-transporting, mitochondrial F1 complex, β polypeptide (ATP5B)*, forward primer 5′-ggcacaatgcaggaaagg-3′, reverse primer 5′-tcagcaggcacatagatagcc-3′, probe #77; *Complement C1q A chain (C1qa)*, forward primer 5′-ggagcatccagtttgatcg-3′, reverse primer 5′-catccctgagaggtctccat-3′, probe #16; *Complement component 3 (C3)*, forward primer 5′-accttacctcggcaagtttct-3′, reverse primer 5′-ttgtagagctgctggtcagg-3′, probe #76; *Inducible nitric oxide synthase (iNOS)*, forward primer 5′-ctttgccacggacgagac-3′, reverse primer 5′-tcattgtactctgagggctga-3′, probe #13; *Myxovirus influenza resistance 1* (*Mx1*), forward primer 5′-ttcaaggatcactcatacttcagc-3′, reverse primer 5′-gggaggtgagctcctcagt-3, probe #53; *Myxovirus influenza resistance 2* (*Mx2*), forward primer 5′-cagttcctctcagtcccaagat-3′, reverse primer 5′-tgcggttgtgagcctctt-3′, probe #11; *Translocator protein (TSPO)*, forward primer 5′-cccttgggtctctacactgg-3′, reverse primer 5′-aagcagaagatcggccaag-3′, probe #21. *ATPase* was used as reference gene and the ΔΔC method was applied using the LightCycler®480 software 1.2.1 for data evaluation.

### 2.6 Immunohistochemistry and image analysis

Eyes were enucleated and fixed in 4% paraformaldehyde (Roti Histofix; Roth; Karlsruhe, Germany) for 2.5 h at room temperature. For retinal flat mounts, eyes were dissected and permeabilized and unspecific antigens then blocked with PERM/Block Buffer (5% NDS, 0.2% BSA, 0.3% Triton X-100 in PBS) overnight at 4 °C. For cryosections, whole eyes were transferred in 30% sucrose for 1 h before embedding in optimal cutting temperature (O.C.T.) compound. 12 μm sections were prepared with a Leica CM3050 S Cryostat (Leica Biosystems; Wetzlar, Germany). Frozen slides were thawed at RT and dehydrated in PBS for 10 min before unspecific antigens were blocked with BLOTTO (1% milk powder, 0.3% Triton X-100 in PBS) for 30 min at RT. Retinal flat mounts and sections were incubated with primary antibody overnight at 4 °C. A 1:500 dilution of anti-ionized calcium-binding adapter molecule 1 (Iba-1) antibody (FUJIFILM Wako Chemicals Europe; Neuss, Germany) was used. Afterwards, retinal flat mounts (1:1500) and sections (1:1000) were incubated with anti-rabbit Alexa 488 secondary antibody for 1 h at room temperature (Invitrogen; Carlsbad, USA). Retinal flat mounts were mounted on microscopic slides and embedded with Vectashield H-1400 (Vector Laboratories; Burlingame, USA). Sections were mounted in Fluoromount-G with Dapi (ThermoFisher Scientific; Waltham, USA). Images of the central retina were taken with a Zeiss Imager M.2 equipped with ApoTome.2 (Oberkochen, Germany). The total number of Iba1-positive cells was counted in 4 images of each retinal flat mount or 5–10 sections and averaged for one retinal n. Counting was performed with the particle analyzer plugin or the multi-point tool of ImageJ 1.52a (Maryland, USA) in flat mounts and cryosections, respectively.

### 2.7 Statistical analysis

All data were plotted and analyzed with GraphPad PRISM 7.04. After normality tests, *in vitro* data were analyzed using Mann-Whitney *U* test. For *in vivo* data Mann-Whitney *U* test, Kruskal-Wallis test with Dunn's multiple comparison, or one-way ANOVA followed by Tukey's multiple comparison post-test was used as indicated (*p < 0.05, **p < 0.01, ***p ≤ 0.001 and ****p < 0.0001). Error bars show mean ± SEM.

## 3 Results

### 3.1 Biological activity of human IFN-β in murine BV-2 cells

**Fig. 1:**
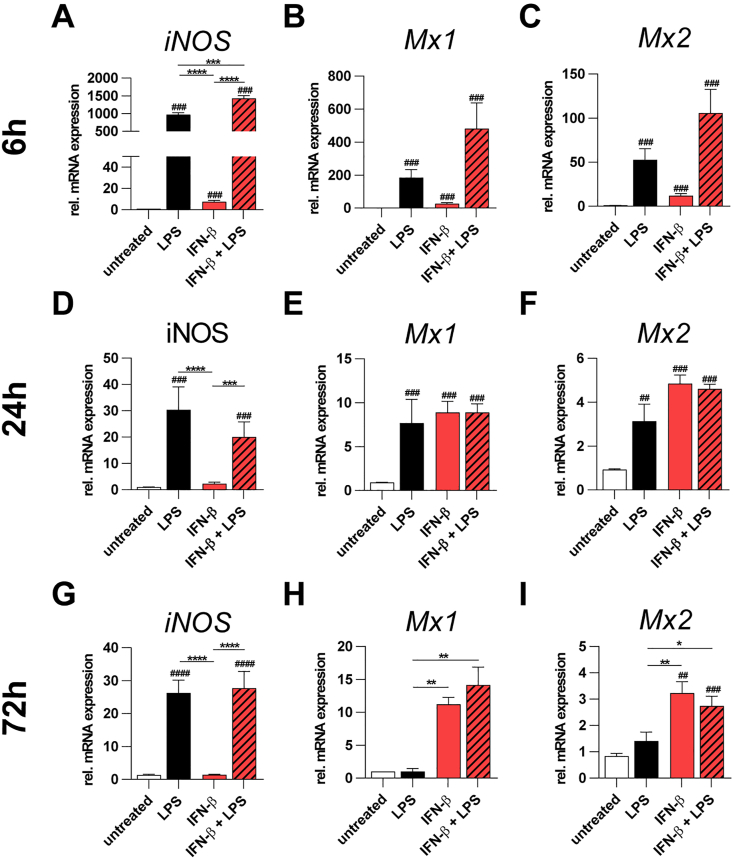
Biological activity of human IFN-β in BV-2 mouse microglia cells. Cells were treated with LPS (50 ng/mL) and IFN-β (1000 U/mL) for 6 h qRT-PCR was performed and ΔΔCT analysis was used for quantification. *ATP5B* was used as reference gene. Graphs were plotted with GraphPad Prism 7.04. Bars represent mean ± SEM. Data were analyzed using Mann-Whitney *U* test. # depicts significance versus control. n = 6–8 (control), n = 6–12 (treated samples).

We first evaluated the biological activity of human recombinant IFN-β in murine BV-2 microglia cells that were previously untreated or simultaneously activated with Lipopolysaccharide (LPS). The mRNA expression of *inducible nitric oxide synthase (iNOS)* severed as a pro-inflammatory control and two well-known IFN-stimulated transcripts, *Myxovirus influenza resistance 1* (*Mx1*) and *2 (Mx2)* [21,24] were analyzed using qRT-PCR (Fig. 1). The BV-2 cells were treated for 6, 24, and 72 h, respectively, to analyze time-dependent mRNA expression levels for these three candidate genes. In the 6 h short term stimulation experiments, all three analyzed transcripts were significantly increased by stimulation with either LPS or IFN-β or both together (Fig. 1A–C). This indicates that the BV-2 cells were sensitive to LPS and IFN-β in this early phase and that both signaling pathways were active. At 24 h after stimulation, only LPS was able to trigger expression of all three genes (Fig. 1D–F), whereas the effect of IFN-β was only seen for the two interferon-responsive genes (Fig. 1E and F). In late phase experiments, LPS could only stimulate *iNOS*, whereas *Mx1* and *Mx2* were exclusively responsive to IFN-β single or co-treatment (Fig. 1G–I). These data indicate that human IFN-β is biologically active in murine microglia cells and that interferon responsive genes *Mx1* and *Mx2* are activated over a longer time span.

### 3.2 IFN-β treatment in a murine light-damage paradigm

We were next interested to study the biological effects of human IFN-β in a murine model of light-induced retinal degeneration. BALB/cJ mice were treated with 10,000 U human IFN-β every other day starting 1 h prior to light exposure.

**Fig. 2:**
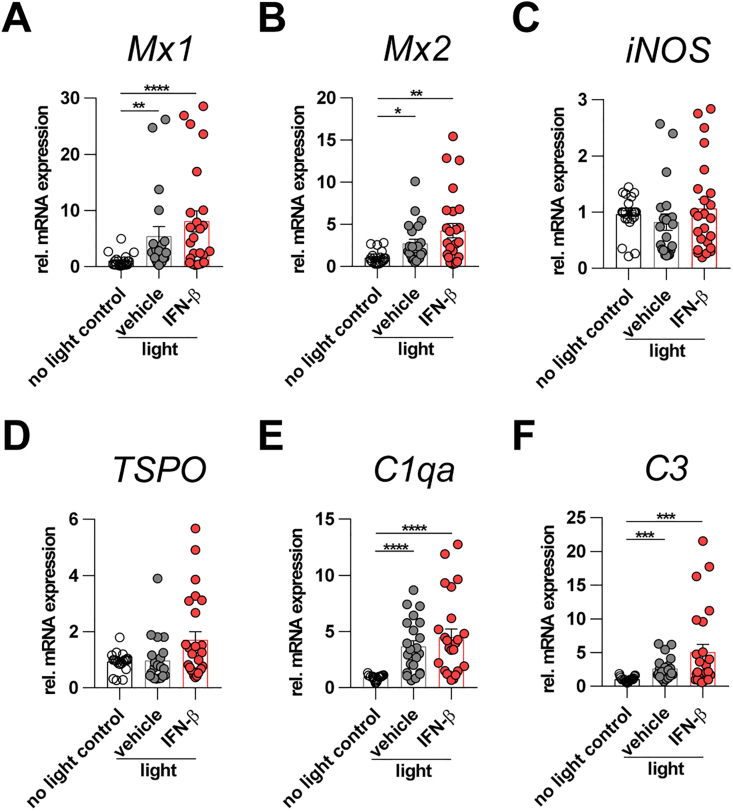
Expression analysis of retinal tissue. Mice were treated with IFN-β (10,000 U) or PBS as vehicle every other day starting 1 h prior to light damage until 4 d afterwards. qRT-PCR was performed and ΔΔCT analysis was used for quantification. *ATP5B* was used as reference gene. Graphs were plotted with GraphPad Prism 7.04. Bars represent Mean ± SEM. Data were analyzed using Kruskal-Wallis test followed by Dunn's multiple comparison. n = 23 (no light control), n = 20–22 (light + vehicle), n = 24–25 (light + IFN-â).

qRT-PCR analysis of retinal tissue was performed to examine changes in expression of IFN-stimulated genes, complement components and inflammation modulators (Fig. 2). In accordance with the cell culture experiments, *Mx1* and *Mx2* expression was significantly upregulated by light damage in both vehicle-treated and IFN-β-treated conditions (Fig. 2 A, B). *iNOS* transcripts and the early microglia marker *translocator protein (TSPO)* were unchanged four days after light exposure (Fig. 2C, D). As was seen for *Mx1* and *Mx2* genes, *complement component 3 (C3)* and *complement C1q A chain (C1qa)* transcripts were significantly upregulated by light damage in both vehicle-treated and IFN-β-treated conditions (Fig. 2 E, F).

These findings implicate that light damage alone already induced IFNAR signaling in the retina and that systemic treatment with human IFN-β showed only a trend towards higher induction levels of IFNAR responsive genes.

**Fig. 3:**
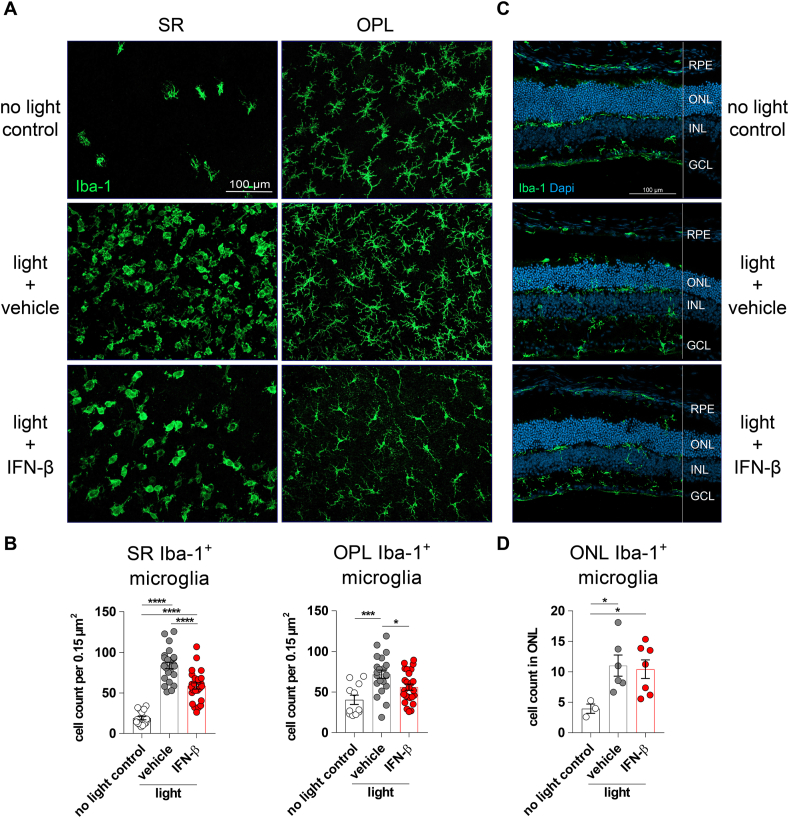
Iba-1 staining of microglia. Mice were treated with IFN-β (10,000 U) or PBS as vehicle every other day starting 1 h prior to light damage until 4 d afterwards. **A** Microglia were stained on retinal flat mounts using ionized calcium-binding adapter molecule 1 (Iba-1). **B** 4 images of the central retina were taken around the optic nerve of each eye and averaged for 1 n. Cell numbers were counted using the particle analyzer plugin of ImageJ. Data were analyzed using one-way ANOVA and Tukey's multiple comparison test. n = 17 (no light), n = 25 (light). **C** Cryosections were stained with DAPI and Iba-1. **D** 5–10 images of the central retina were taken of each eye and averaged for 1 n. Cell numbers were counted using the multi-point tool of ImageJ. Data were statistically analyzed using the Mann-Whitney *U* test. n = 3 (no light control), n = 6 (light + vehicle), n = 7 (light + IFN-β). **B, D** Graphs were plotted with GraphPad Prism 7.04. Bars represent mean ± SEM. SR: subretinal space; OPL: outer plexiform layer; RPE: retinal pigment epithelium; ONL: outer nuclear layer; INL: inner nuclear layer; GCL: ganglion cell layer.

We next performed a systematic morphometric analysis of retinal microglia behavior. Therefore, microglia numbers, location, and morphology were determined using retinal flat mounts and cryosections that were stained with Iba-1 (Fig. 3). Mainly ramified microglia were detected in the outer plexiform layer and only few cells were present in the subretinal space of control animals (Fig. 3 A). After light exposure, microglia were activated and recruited to the subretinal space. Interestingly, IFN-β treatment resulted in significantly less amoeboid microglia numbers in the subretinal space and in the outer plexiform layer compared to vehicle-treated animals (Fig. 3 B). These findings were also confirmed by Iba-1 stained cryosections, where less amoeboid microglia were present in the outer plexiform layer and in the subretinal space after IFN-β therapy compared to the vehicle group (Fig. 3C). However, a reduction of infiltrating microglia numbers in the outer nuclear layer could not be observed (Fig. 3 D).

**Fig. 4:**
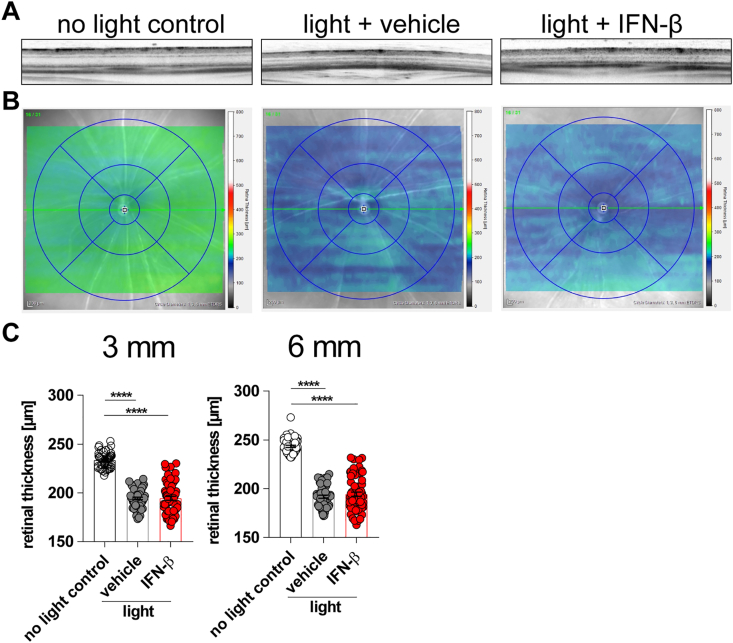
Retinal thickness analysis using SD-OCT. Mice were treated with IFN-β (10,000 U) or PBS as vehicle every other day starting 1 h prior to light damage until 4 d afterwards. **A** Representative scans and **B** corresponding heatmaps compiled by spectral-domain optical coherence tomography (SD-OCT) displaying a retinal overview. **C** Retinal thickness was plotted with GraphPad Prism 7.04. Bars represent Mean ± SEM. Data were analyzed using one-way ANOVA and Tukey's multiple comparison test. n = 55 (no light control), n = 57 (light + vehicle), n = 63 (light + IFN-β).

The DAPI-staining in Fig. 3C already indicated that the light-damage induced thinning of the outer nuclear layer was not affected by IFN-β treatment. We therefore quantified the overall retinal thickness in these experimental groups using SD-OCT scans (Fig. 4). These scans indicated that light damage changed the overall reflectance patterns (Fig. 4 A). As clearly seen from the retinal heatmaps of individual animals (Fig. 4 B) and combined analyses of larger animal numbers (Fig. 4C), a significant reduction of retinal thickness was observed after light damage and this was not influenced by IFN-β therapy.

## 4 Discussion

Type I IFNs are known to play an important role in activating the innate and adaptive immune response, but excessive IFN signaling has been implicated in the pathogenesis of several diseases [25]. Here we showed that IFN-β is a potent immunomodulator of retinal microglia activity but is not preventing from photoreceptor cell loss in an experimental mouse model of dry AMD.

IFN-β signaling has several complex effects [26]. In a mouse model of experimental autoimmune encephalomyelitis (EAE), ablation of IFNAR or IFN-β genes resulted in exacerbated chronicity of the clinical disease, accompanied by extensive microglia activation and inflammatory response [20,27]. Engagement of myeloid-specific IFNAR by locally produced IFN-β reduced inflammation in effector phases [20]. Nonetheless, the number of infiltrating mononuclear cells was not altered in this experimental setting [20]. These results from the brain are somewhat contradictory to our findings in the retina, since we detected less mononuclear phagocytes into the subretinal space. In line with that, IFN-β can dose-dependently reduce the expression of MHC class II molecules on murine macrophages [28] and microglia [29], as well as human monocytic cells [30]. A possible mechanism of immune modulation by IFN-β via antigen presentation could be interference with T cell activation. Indeed, there is evidence that both innate and adaptive immune mechanisms are linked to AMD pathogenesis [31].

The effects of IFN-β on microglia depend on mode and conditions of activation [32]. Likewise, IFN-β did not prevent from neuronal cell death despite its suppression of glutamate and superoxide production [32]. IFN-β blocked the expression of cytotoxins (tumor necrosis factor α and lymphotoxin) by stimulating human blood mononuclear cells, albeit with large interindividual variations in response to the suppressive effects of IFN-β [33]. Hence, variability in IFN-β effects could be due to different species, source of recombinant IFN-β and experimental setup and thus needs clarification with further research. Nonetheless, local immunomodulation in the retina has been shown to be effective in another light damage paradigm. Thus, intravitreal injection of cerium oxide nanoparticles showed a full rescue of the retinal phenotype and function via reduction of microglia activation and reactive oxygen species [34], while intravenous injection was not affective [35].

IFN-β is clinically used for multiple sclerosis therapy since 1993 [15], modulating antigen presentation and cytokine expression patterns. Dose and frequency of IFN-β administration affect its efficacy in patients [36]. Higher doses at a more frequent level decreased the number of relapses in patients. However, raising dose and/or injection frequency did not yield different results in our study (data not shown). Interestingly, earlier studies of our lab showed that IFN-β treatment attenuated microglia reactivity and significantly reduced CNV size in a laser-induced mouse model for wet AMD [21]. Despite the use of C57BL/6 mice in the previous study, no difference in the *Mx* response of retinal tissue was seen (data not shown), eliminating potential influences of the genetic background. However, a recent study found differences in alternative complement component expression between C57BL/6 and BALB/c mouse strains [37].

In summary, our study showed that IFNAR signaling in the retina significantly reduced the number of reactivate microglia in the subretinal space of light-damaged mice. However, neither a decreased expression of complement factors nor a rescue of retinal thickness was detectable after IFN-β therapy.

## Funding

This work was supported by the Helmut Ecker Foundation [03/17]; the 10.13039/501100001659Deutsche Forschungsgemeinschaft [FOR2240, Project 6]; and the Center for Molecular Medicine Cologne.

## Author statement

VB participated in the design of experiments, conducted the experiments, analyzed and interpreted the data, wrote the first manuscript draft, and assembled the figures. TL developed the study concept and all experimental designs, supervised the project, and wrote and revised the manuscript. The authors read and approved the final manuscript.

## Declaration of competing interest

All authors declare no conflict of interest.
